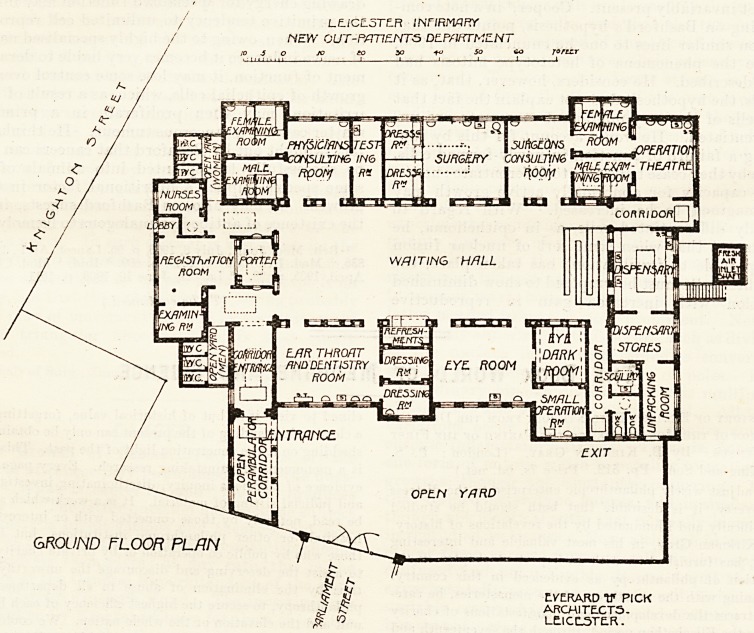# Out-Patients' Department, Leicester Infirmary

**Published:** 1906-01-27

**Authors:** 


### HOSPITAL ADMINISTRATION.

### CONSTRUCTION AND ECONOMICS.

### OUT-PATIENTS' DEPARTMENT, LEICESTER INFIRMARY.

The old out-patients' department occupied the ground floor of the south-west wing of the main building, an arrangement which necessarily entailed more or less communication between the in-patients and the out-patients; and, moreover, the old department was in many respects faulty. Now an entirely new and separate block has been erected for the out-patients.

hall are the doors of the physician's consulting room, opening from which are the men and women's examining rooms and the testing room. Then the surgery is reached with its dressing-rooms and surgeon's consulting room, adjoining which is the operating theatre. The latter has an additional door opening into a corridor which communicates with the dispensary and with the waiting hall. The dispensary thus occupies the end of the hall opposite the porter's room and

The entrance is in Parliament Street. This first of all leads into an open yard, on the left hand of which is a large, partly open corridor, one end of which is used for perambulators and the other leads into a lobby, where are the registration room, examining room, nurses' room, closets for men and women, and the porter's room. These take up the Knighton Street end of the building. Patients pass along from the porter's room to the waiting-room, which is really a splendid hall 85 feet long and 29 feet wide; and is centrally placed, having grouped around it the offices already named and those to be presently mentioned.

Beginning at the left-hand corner, on entering the waiting entrance. Near the dispensary is a corridor leading to the exit door. Beyond the hall end of the corridor is the eye department, having a dark chamber and a small operating theatre. Then there is a common room for the ear, throat, and dentistry. The refreshment buffet is also here. It seems certain that the whole of this out-patients' department is the outcome of a carefully thought out scheme. All the arrangements look satisfactory on plan, and they should be equally as satisfactory in practical working.

The block is warmed on the Plenum vaccum system.

The architects were Messrs. Everard and Pick; and the contractor was Mr. Charles Wright.

## Figures and Tables

**Figure f1:**